# Substance P and Glucagon-like Peptide-1_7-36_ Amide Mediate Anorexic Responses to Trichothecene Deoxynivalenol and Its Congeners

**DOI:** 10.3390/toxins14120885

**Published:** 2022-12-18

**Authors:** Hui Jia, Zihui Qin, Ben Wei, Xinyi Guo, Huiping Xiao, Huayue Zhang, Zelin Li, Qinghua Wu, Ruibo Zheng, Wenda Wu

**Affiliations:** 1 School of Animal Husbandry and Veterinary Medicine, Jiangsu Vocational College of Agriculture and Forestry, Jurong 212400, China; 2 School of Food and Biological Engineering, Hefei University of Technology, Hefei 230009, China; 3 MOE Joint International Research Laboratory of Animal Health and Food Safety, College of Veterinary Medicine, Nanjing Agricultural University, Nanjing 210095, China; 4 Department of Chemistry, Faculty of Science, University of Hradec Kralove, 50003 Hradec Kralove, Czech Republic; 5 College of Life Science, Yangtze University, Jingzhou 434025, China

**Keywords:** trichothecene, anorexia, brain-gut peptide, substance P, glucagon-like peptide-1_7-36_ amide

## Abstract

Type B trichothecenes commonly contaminate cereal grains and include five structurally related congeners: deoxynivalenol (DON), 3-acetyldeoxynivalenol (3-ADON), 15-acetyldeoxynivalenol (15-ADON), fusarenon X (FX), and nivalenol (NIV). These toxins are known to have negative effects on human and animal health, particularly affecting food intake. However, the pathophysiological basis for anorexic effect is not fully clarified. The purpose of this study is to explore the potential roles of the brain-gut peptides substance P (SP) and glucagon-like peptide-1_7-36_ amide (GLP-1) in anorexic responses induced by type B trichothecenes following both intraperitoneal (IP) and oral administration. SP and GLP-1 were elevated at 1 or 2 h and returned to basal levels at 6 h following exposure to DON and both ADONs. FX induced the production of both brain gut peptides with initial time at 1 or 2 h and duration > 6 h. Similar to FX, exposing IP to NIV caused elevations of SP and GLP-1 at 1 h and lasted more than 6 h, whereas oral exposure to NIV only increased both brain gut peptides at 2 h. The neurokinin-1 receptor (NK-1R) antagonist Emend^®^ dose-dependently attenuated both SP- and DON-induced anorexic responses. Pretreatment with the GLP-1 receptor (GLP-1R) antagonist Exending_9-39_ induced a dose-dependent attenuation of both GLP-1- and DON-induced anorexic responses. To summarize, the results suggest that both SP and GLP-1 play important roles in anorexia induction by type B trichothecenes.

## 1. Introduction

Type B trichothecenes constitute a class of mycotoxin frequently detected in cereals and grain products. Deoxynivalenol (DON) is the most commonly researched mycotoxin in the field of food safety. Its four structurally-related congeners 3-acetyldeoxynivalenol (3-ADON), 15-acetyldeoxynivalenol (15-ADON), fusarenon X (FX), and nivalenol (NIV) are also regarded as important risk factors [1]. Many adverse effects caused by this family have been reported, such as anorexia, emesis, growth suppression, neuroendocrine changes, cytotoxicity, immunotoxicity, the inhibition of protein synthesis and mitochondrial translation [2,3,4,5]. DON and its congeners are well- known for causing decreased feed intake in human and a variety of animals [6,7,8,9] including broilers, pigs, dairy cow, mice and cats. The mechanism of type B trichothecene-induced anorexia is still not entirely clear.

Food intake is influenced by both central factors and peripheral factors [10,11]. Anorexigenic molecules involving pro-opiomelanocortin (POMC), cocaine and amphetamine-regulated transcript (CART), melanocortin-3 receptor (MC3R), melanocortin-4 receptor (MC4R) and orexigenic molecule involving neuropeptide Y (NPY), and agouti-related peptide (AgPR) regulate appetite together. DON-induced anorexia is partly due to the up-regulation of central anorexigenic factors and the massive release of the peripheral satiety hormones, such as peptide YY(PYY) [12,13]. In addition, the release of some neurotransmitters located in the gastrointestinal (GI) tract play an important role in the regulation of anorexigenic signaling [14]. Our previous studies have shown that DON mediates anorexia by promoting the secretion of the neurotransmitters GLP-1 and GIP [15]. It has also been found that T2 toxins mediate anorexia by activating the secretion of the peptide neurotransmitter SP. Coincidentally, the secretion of both SP and GLP-1 can up-regulate the central anorexigenic factors [16]. However, the role of these two peptide neurotransmitters in type B trichothecenes-induced anorexia is not clear.

SP is an undecapeptide of the neurokinin family, which abounds densely within nucleus tractus solitarii (NTS),the vagal and enteric nervous systems [17,18,19]. The food intake suppression of SP is involved in modulating the CRH signal and upregulating POMC expression [20,21,22]. GLP-1 is produced primarily by L cells in the distal ileum and colon, and is considered to action both as a peripheral satiety hormone and as a central neurotransmitter [23]. Studies of GLP-1 have expanded beyond glucose control to appetite regulation, energy balance and many more functions. The purpose of this study was to test the hypothesis that type B trichothecenes induce the release of SP and GLP-1 in mice and determine the role of these brain gut peptides in the anorectic effect of type B trichothecenes. Here, a proven murine anorexia model was used to relate SP and GLP-1 plasma concentrations using different methods (oral vs. IP) to five type B trichothecenes-induced food refusal. The SP and GLP-1 receptor antagonists were employed to verify the anorectic response.

## 2. Results

**Figure 1:**
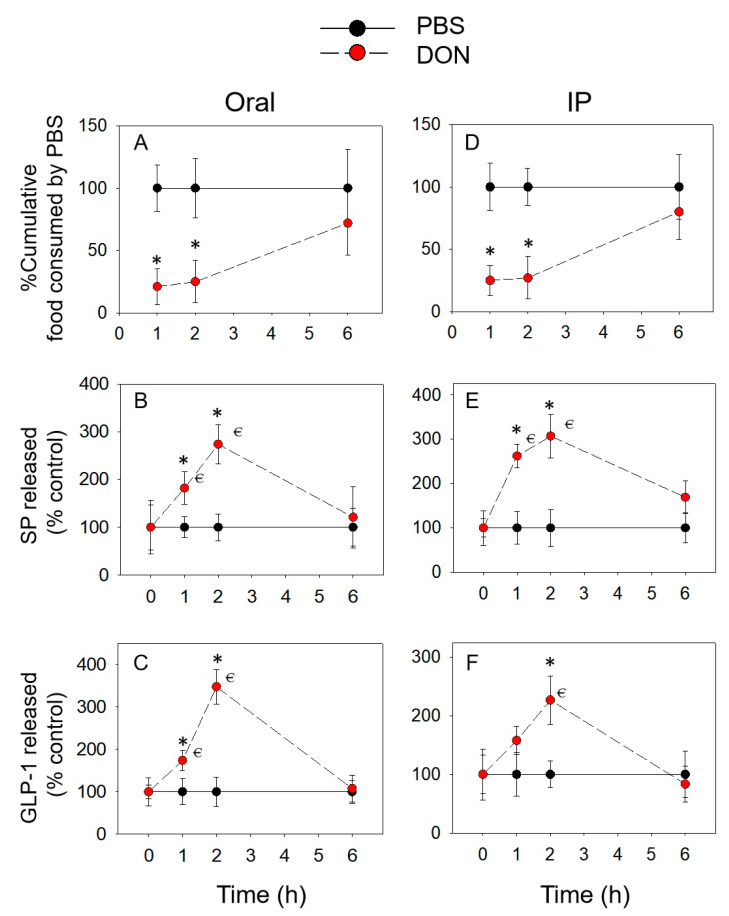
DON-induced anorexic responses (**A**,**D**) correspond to the elevation of plasma SP (**B**,**E**) and GLP-1 (**C**,**F**). Mice were administered orally (**A**–**C**) and intraperitoneally (**D**–**F**) with PBS (solid lines) or DON (broken lines). * indicates a statistically significant difference relative to the control at a specific time point (*p* < 0.05). ⋲ indicates a statistically significant difference relative to the 0 h time point (*p* < 0.05).

After the oral and IP administration of DON, feed intake decreased significantly at 1 h (79% and 74%) and 2 h (75% and 69%), and showed a trend of recovery at 2–6 h after exposure, respectively (Figure 1A,D). Plasma SP concentration increased at 1 and 2 h, and returned to the initial concentration after 6 h (Figure 1B,E). Whereas, GLP-1 was only upregulated markedly at 2 h following IP exposure (Figure 1C,F).

**Figure 2:**
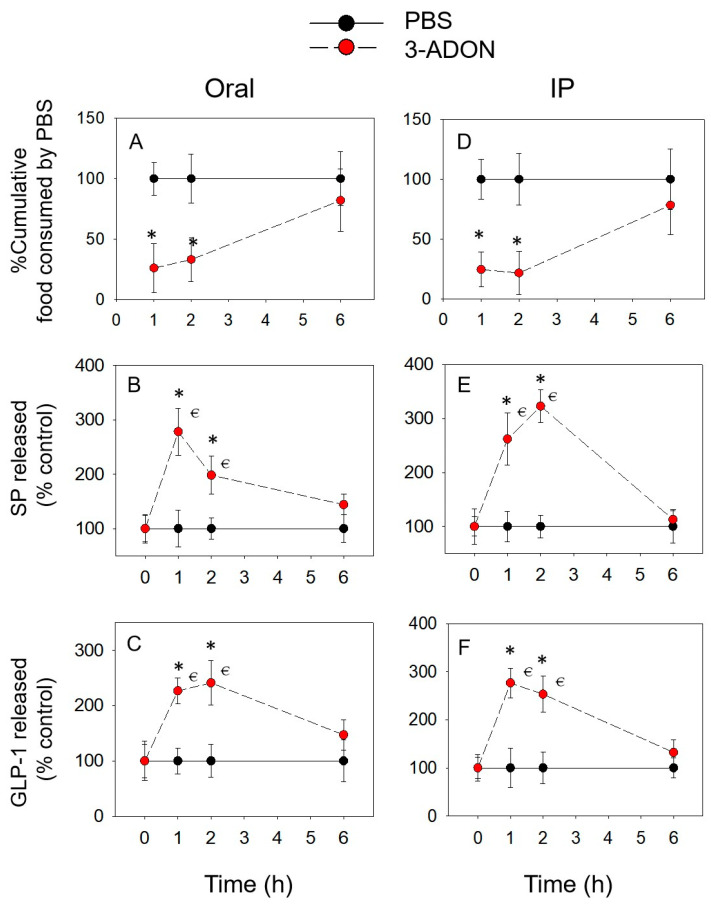
3-ADON-induced anorexic responses (**A**,**D**) correspond to the elevation of plasma SP (**B**,**E**) and GLP-1 (**C**,**F**). * indicates a statistically significant difference relative to the control at a specific time point (*p* < 0.05). ⋲ indicates a statistically significant difference relative to the 0 h time point (*p* < 0.05).

Food consumption was reduced significantly after the oral and IP administration of 3-ADON, e.g., DON at 1 h (72% and 75%) and 2 h (67% and 80%), and recovered at 6 h, respectively (Figure 2A,D). The concentrations of plasma SP (Figure 2B,E) and GLP-1 (Figure 2C,F) were significantly elevated by 3-ADON at 1 and 2 h, but returned to normal at 6 h.

**Figure 3:**
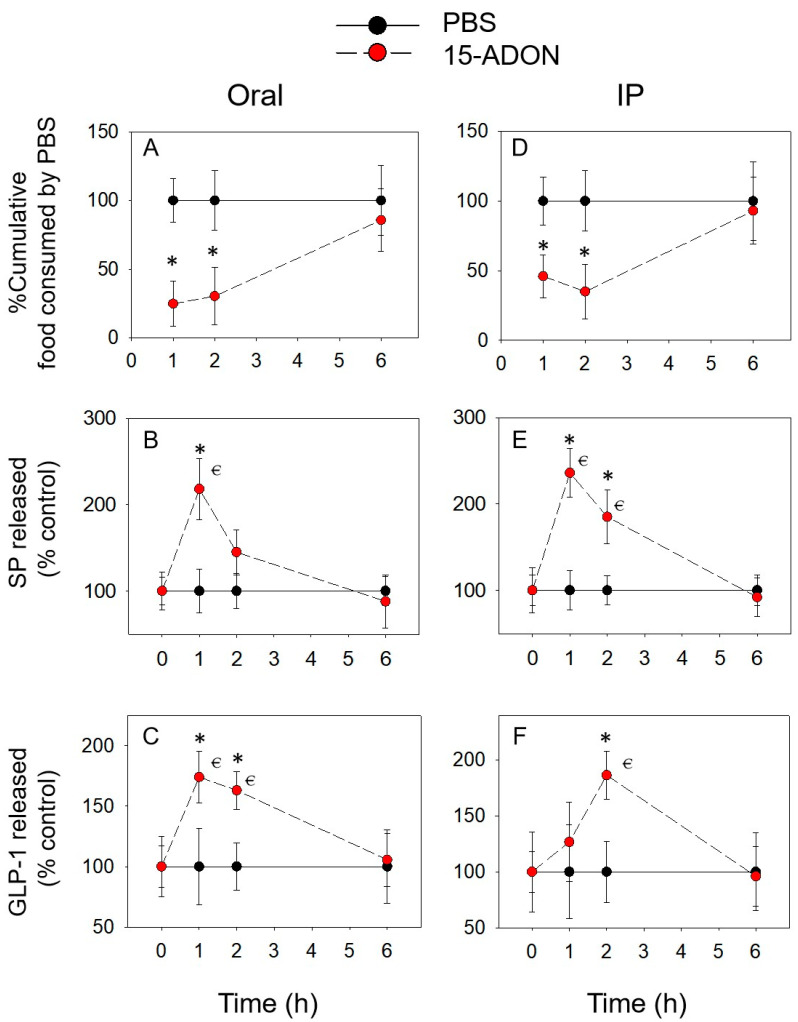
15-ADON-induced anorexic responses (**A**,**D**) correspond to elevation of plasma SP (**B**,**E**) and GLP-1 (**C**,**F**). * indicates a statistically significant difference relative to the control at a specific time point (*p* < 0.05). ⋲ indicates a statistically significant difference relative to the 0 h time point (*p* < 0.05).

Additionally, 15-ADON induced anorexic responses similar to DON, with the feed intake significantly reduced at 1 h (76% and 54%) and 2 h (68% and 63%), and recovered at 6 h, with both oral and IP administration of 15-ADON (Figure 3A,D). Different from DON, plasma SP was elevated only at 2 h following oral exposure (Figure 3B,E). However, IP exposed to 15-ADON induced an increase in SP at 1 and 2 h. After 6 h, no difference in SP was observed. In contrast, plasma GLP-1 (Figure 3C,F) was significantly elevated by 3-ADON at 1 and 2 h after oral exposure. The concentration of plasma GLP-1 was robust upregulated only at 2 h through IP administration.

**Figure 4:**
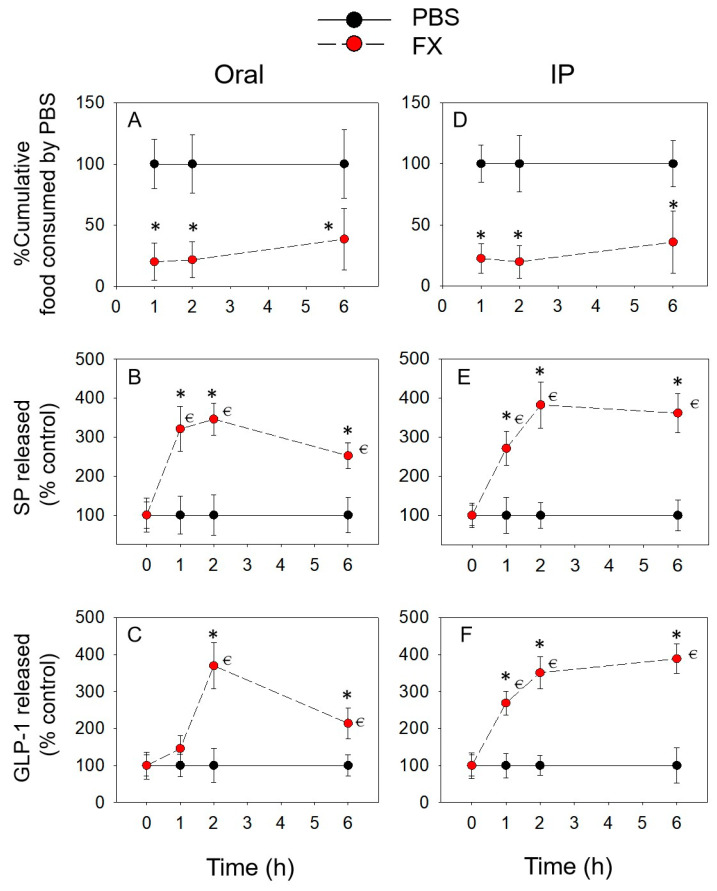
FX-induced anorexic responses (**A**,**D**) correspond to elevation of plasma SP (**B**,**E**) and GLP-1 (**C**,**F**). * indicates a statistically significant difference relative to the control at a specific time point (*p* < 0.05). ⋲ indicates a statistically significant difference relative to the 0 h time point (*p* < 0.05).

FX caused rapid and prolonged (more than 6 h) anorexic responses following both oral and IP treatments (Figure 4A,D). Plasma SP (Figure 4B,E) was raised at 1 h and 2 h and still upregulated at 6 h. The concentration of plasma GLP-1 was increased at 2 and 6 h after oral exposure (Figure 4C,F), whereas IP exposed to FX evoked caused GLP-1 release at 1, 2 and 6 h. The prolonged elevations of SP and GLP-1 correlated with a persistent anorexic effect induced by FX.

**Figure 5:**
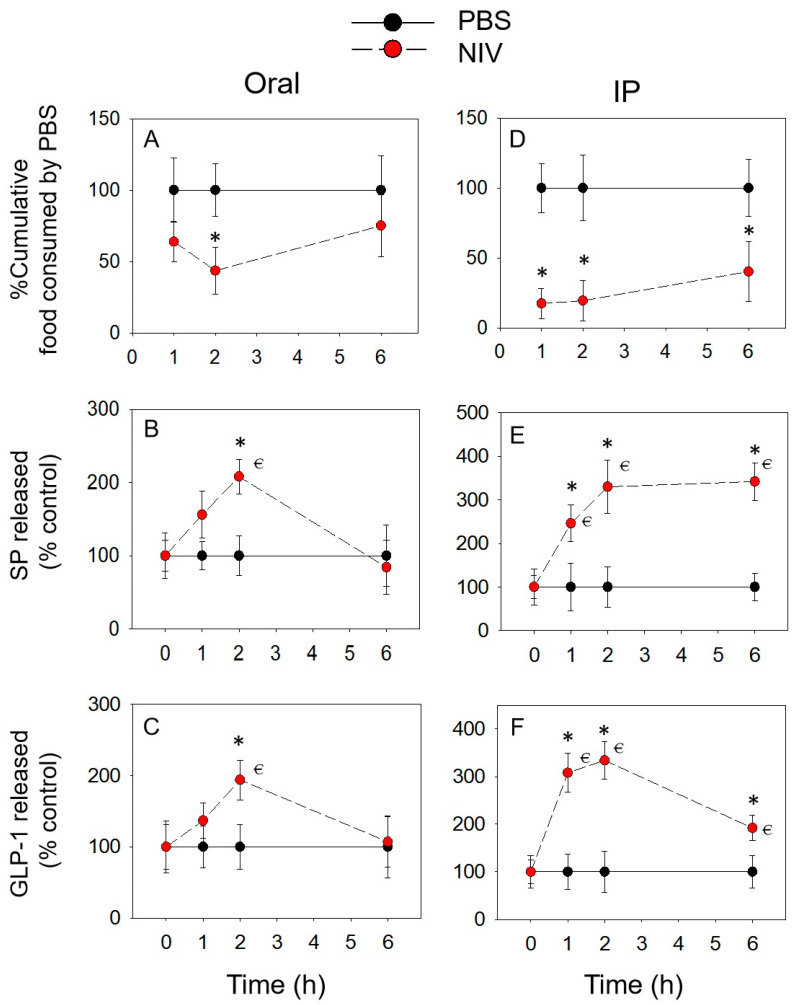
NIV-induced anorexic responses (**A**,**D**) correspond to elevation of plasma SP (**B**,**E**) and GLP-1 (**C**,**F**). * indicates a statistically significant difference relative to the control at a specific time point (*p* < 0.05). ⋲ indicates a statistically significant difference relative to the 0 h time point (*p* < 0.05).

Unlike DON, NIV induced significant anorexia only at 2 h (56%) by oral administration (Figure 5A). Interestingly, plasma SP and GLP-1 concentrations increased significantly only at 2 h, consistent with the anorexic response (Figure 5B,C). After the IP administration of NIV, feed intake decreased significantly at 1 h (83%), 2 h (80%), and 6 h (59%) (Figure 5D). The concentration of plasma SP and GLP-1 also increased significantly at 1, 2 and 6 h (Figure 5E,F).

**Figure 6:**
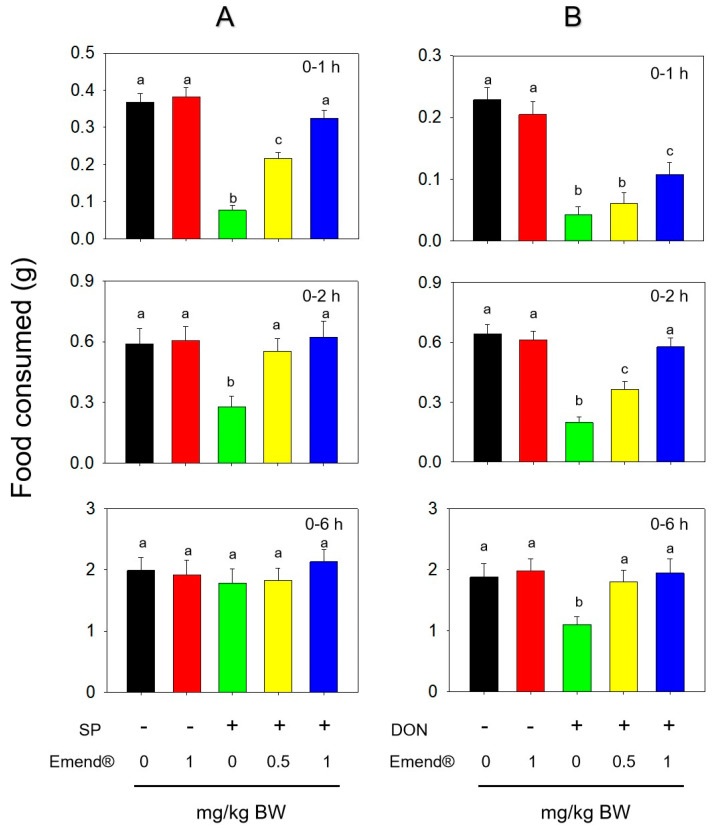
NK−1 receptor antagonist Emend^®^ inhibits (**A**) SP− and (**B**) DON−induced anorexic responses. Bars without the same letter are significantly different (*p* < 0.05).

Emend^®^, a receptor antagonist of NK-1R, was used to study SP- and DON-induced anorexia. Feed intake was significantly reduced at 1 h and 2 h after the IP administration of SP with 0.5 mg/kg BW and returned to normal at 6 h (Figure 6A). Serious anorexia occurred at 1 h, 2 h and 6 h after the IP administration of DON at 2.5 mg/kg BW (Figure 6B). Exposure to Emend^®^ (1 mg/kg BW) alone had no effect on food consumption. Emend^®^ attenuated SP- and DON-induced food refusal in a dose-dependent manner. Following the pretreatment of Emend^®^ at 0.5 mg/kg BW, mice exposed to DON consumed 9, 26 and 37% more food at 1, 2 and 6 h, respectively. Mice receiving 1 mg/kg BW Emend^®^ consumed 28, 59 and 45% more food at 1, 2 and 6 h than DON alone, respectively.

**Figure 7:**
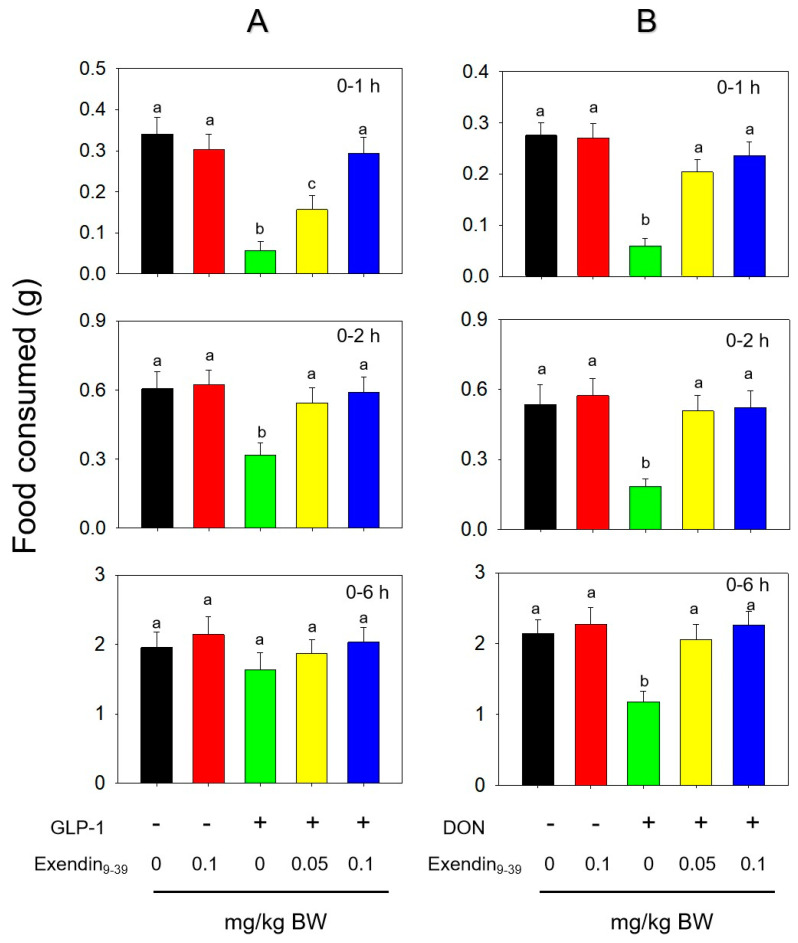
The GLP−1 receptor antagonist Exending_9-39_ inhibits (**A**) GLP−1- and (**B**) DON−induced anorexic response. Bars without the same letter are significantly different (*p* < 0.05).

Exending_9-39_, a receptor antagonist of GLP-1R, was used to study GLP-1- and DON- induced anorexia. Feed intake was significantly reduced at 1 h and 2 h after the IP administration of GLP-1 at 0.25 mg/kg BW and returned to normal at 6 h (Figure 7A). Serious anorexia occurred at 1 h, 2 h and 6 h after the IP administration of DON with 2.5 mg/kg BW (Figure 7B). Exposure to Exending_9-39_ (0.1 mg/kg BW) alone had no effect on food consumption. Exending_9-39_ attenuated SP- and DON- induced food refusal in a dose-dependent manner. Following the pretreatment of Exending_9-39_ at 0.05 mg/kg BW, mice exposed to DON consumed 52, 61 and 41% more food at 1, 2 and 6 h, respectively. Mice pretreated with 0.1 mg/kg bw Exending_9-39_ consumed 61, 64 and 50% more food at 0.5, 2 and 6 h than DON alone, respectively.

## 3. Discussion

Mycotoxins in food and feed continue to threaten the health of humans and animals [24]. It is conservatively estimated that 25% of global food crops are contaminated with mycotoxins [25,26]. Type B trichothecenes are common in cereals. The mechanism behind the toxicity of DON, a type B trichothecene, has been extensively studied, but its four derivatives are often neglected. We integrated the data of trichothecene-induced anorexia of five type B trichothecenes in mice, and compared the changes in plasma concentrations of SP and GLP-1 with different administration methods (oral vs. IP). Additionally, DON-induced anorexia was inhibited by the SP receptor (NK-1 receptor) antagonist Emend^®^ and the GLP-1 receptor antagonist Exendin9-39, confirming both brain-gut peptides may contribute to type B trichothecenes-induced food refusal in mice.

Food consumption were markedly reduced with the IP administration of five type B trichothecenes. The same anorexia responses were observed after the oral administration of DON,3-ADON and 15-ADON; NIV was slightly different in that feed intake decreased significantly at 2 h, although there was also a downward trend in feed intake at 1 h compared with the control group after oral administration [27]. Consistent with previous research, five type B trichothecene-induced anorexia responses disappeared rapidly. The possible reason for this is that most neurotransmitters or satiety hormones which regulate appetite, are short-term [28,29].

The plasma concentrations of SP with IP administration (five type B trichothecenes) and oral administration (DON,3-ADON, FX) were increased significantly at 1 h and 2 h. Moreover, 15-ADON and NIV, when orally administered, were a little different from the others; plasma SP increased significantly at 1 h and 2 h. SP is found in various tissues of the body [30] and the SP-induced anorectic response has been demonstrated [31]. Coincidentally, SP was first isolated from equine brain and gut [32]. Appetite and vomiting are regulated by SP through the central nervous system [33]. SP can also be secreted by gastrointestinal EC cells and bind to neurokinin 1 receptor (NK-1R) in the abdominal vagal nerve through paracrine, sending satiety signals via the brain-gut axis. The hypothalamus receives the signals and regulates appetite [34]. There is another interesting finding; SP is known as an antagonist of the GHSR-la receptor which inhibits ghrelin-induced food intake [35]. We hypothesize that five type B trichothecenes may promote gastrointestinal SP secretion to regulate feed intake.

GLP-1 is released mostly by the L-cells of the intestines and is known for regulating energy balance (glucose homeostasis and appetite) via the peripheral and central systems [36,37]. The main function of GLP-1 is to inhibit the release of glucagon and stimulate the release of insulin [38]. An injection of GLP-1 into the lateral hypothalamus (LH), the core area for food intake, reduces food intake [39]. GLP-1 affects feed intake by regulating dopamine synthesis, the glutamate neurotransmitter and c-fos immunoreactivity [40,41,42]. The plasma concentrations of GLP-1 with oral administration and IP administration were increased significantly. Plasma GLP-1 with IP administration (DON, 15-ADON) and oral administration (FX, NIV) were increased significantly at 2 h. In addition, various bitter substances such as quinine, berberine and gentian root extract promote GLP-1 secretion by activating bitter taste receptor pathways [43,44,45]. Bitter substances are divided into five categories: terpenes and steroids, inorganic salts, alkaloids, flavanone glycosides, amino acids and peptides. These five type B trichothecenes belong to the terpene family. Therefore, we hypothesized that these five type B trichothecene-induced anorexia responses also promote GLP-1 release by activating the bitter taste receptor signaling pathway in the gut.

Neurokinin receptors belong to the family of seven-transmembrane, G protein-coupled receptors, including NK1R, NK2R and NK3R, with SP preferring NK1R [46,47]. GLP-1 receptors are widely distributed in the hypothalamus, the vagal afferents, the area postrema (AP), the NTS and the intestine [48]. We found here that exogenous SP and GLP-1 induced food refusals, which could be inhibited by their antagonists, Emend^®^ and Exendin9–39 [49,50]. NK1R and GLP-1R play an important role in DON-induced anorexia.

## 4. Conclusions

Our findings here suggest that five type B trichothecenes induce food refusal, which is consistent with plasma SP and GLP-1 elevation, activating both NK-1R and GLP-1R. Previous studies found that five type B trichothecenes alter the brain-gut systems via the release of neurotransmitters and gut hormones, such as 5-hydroxytryptamine, PYY and gastric inhibitory peptide. Future studies should aim to determine the molecular mechanism of the type B trichothecene-induced gut enteroendocrine cell secretion of hormones and neurotransmitters. In addition, it is also interesting to explore the role of bitter receptor signaling pathways in mycotoxin-induced anorexia. From an animal and human health perspective, this study has the capability to further improve the understanding of how trichothecene DON and its congeners cause food poisoning and formulate strategies to prevent these adverse effects in the near future.

## 5. Materials and Methods

### 5.1. Animal and Reagent

B6C3F1 mice (10–11 weeks, female, Comparative Medicine Center of Yangzhou University) were individually housed in the room with a normal 12 h light/dark cycle. The room temperature and relative humidity were 20–23 °C and 40–60%, respectively. All guidelines for animal experiments followed the Institutional Animal Care and Use Committee at Nanjing Agricultural University. Type B trichothecenes were tested using a Liquid Chromatograph Mass Spectrometer and by conducting Elemental Analysis with a purity of more than 98%. SP (R&D Systems, Inc), GLP-1 and GLP-1R antagonist Exendin_9-39_ (Sigma-Aldrich, St. Louis, MO, USA) were dissolved in PBS. The NK-1R antagonist Emend^®^ (Merck & Co, Inc) was dissolved in 1% DMSO in filter-sterilized PBS.

### 5.2. Experimental Design

**Figure 8:**
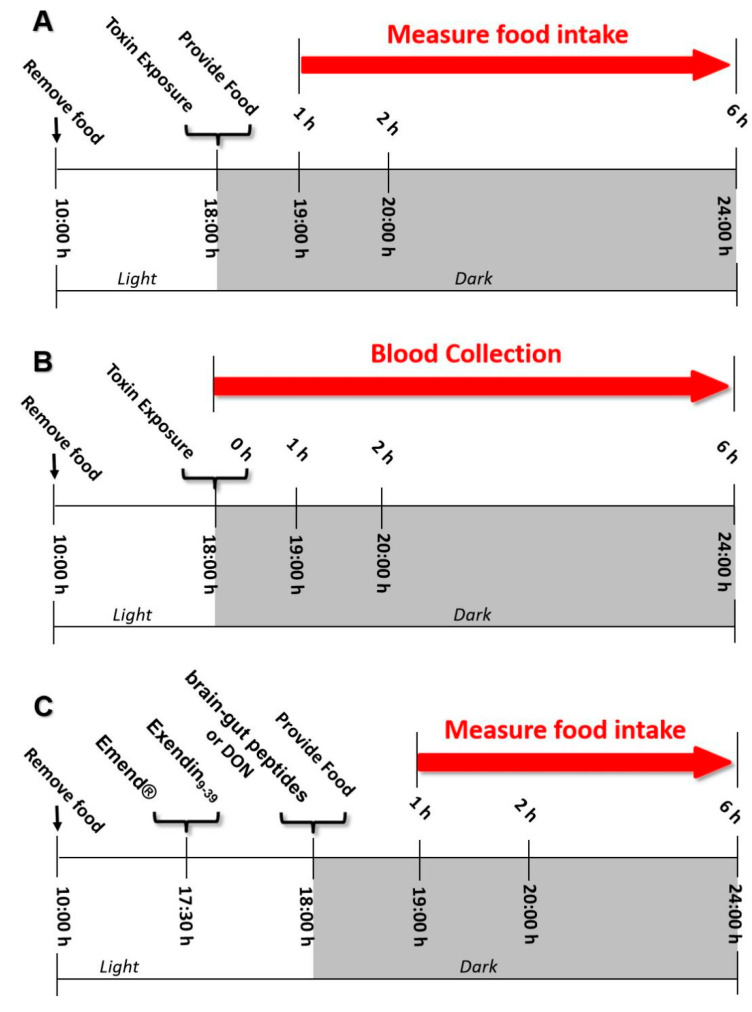
(**A**) Experimental design for anorexia studies. (**B**) Experimental design for brain-gut peptide studies. (**C**) Experimental design for brain-gut peptide receptor inhibitor study.

The design of the experiment with feed intake is shown in Figure 8A. The mice were randomly divided into groups based on body weight with 8 mice in each group and then acclimated to the environment for a week. The food of the mice was removed at 10:00 and the mice were fasted for 8 h on the day of the experiment. Then, 100 µL PBS or triclosporene B was gavaged orally or injected intraperitoneally with a dose of 2.5 mg/kg BW. The mice were given food pellets immediately and food intake was measured at 1, 2 and 6 h.

The design of the study of brain–gut peptides is shown in Figure 8B. Groups of mice (*n* = 8/group) were orally or intraperitoneally administered 100 µL either PBS or type B trichothenenes at 2.5 mg/kg BW, respectively. Then, mice were anesthetized with sodium pentobarbital and sacrificed at 0, 1, 2 and 6 h. Plasma was collected through an EDTA anticoagulant tube and centrifuged for 10 min (3500× *g*, 4 °C). Brain–gut peptides SP and GLP-1 were determined by enzyme-linked immunosorbent assay (Phoenix Pharmaceutical).

The experimental design for this study of brain–gut peptide receptor inhibitors is characterized in Figure 8C. Mice (*n* = 8/group) were orally given 100 μL of NK-1R antagonist Emend^®^ or GLP-1R antagonist Exendin_9-39_ with 0, 0.5 and 1 mg/kg BW or 0, 0.05 and 0.1 mg/kg BW, respectively. After half an hour, mice were administrated an IP injection of SP or GLP-1 at 0.5 or 0.25 mg/kg BW in 100 μL, respectively. Control groups were first administrated either vehicle (1% DMSO) or antagonists orally (1 mg/kg BW Emend^®^ or 0.1 mg/kg BW Exendin_9-39_), and then IP injected with PBS. Food intake was measured at 1, 2 and 6 h post-treatment.

To determine the role of SP or GLP-1 in DON and its congener-induced anorexia, mice were administrated 100 μL of antagonist orally (0, 0.5 and 1 mg/kg BW Emend^®^ or 0, 0.05 and 0.1 mg/kg BW Exendin_9-39_) 30 min before oral exposure to 100 μL of DON at 2.5 mg/kg BW. Controls and food intake measurement time points were the same as the antagonist’s effect on SP and GLP-1-induced food refusal. 

### 5.3. Statistics

All data were analyzed using SigmaPlot 11 for Windows (Jandel Scientific; San Rafael, CA, USA). Data are mean ± SEM (*n* = 8/group). Two-way repeated ANOVA (one factor) using the Holm–Sidak method was used to assess significant differences in food intake compared with the control at specific time points. Two-way ANOVA using Bonferroni t-test was used to analyze significant differences in the kinetics of SP and GLP-1 concentrations in plasma relative to the 0 h time point. One-way ANOVA using the Student–Newman–Keuls method was used to assess significant differences in food intake to determine the role of SP or GLP-1 in DON and its congener-induced anorexia. Significant differences were considered at *p* < 0.05.

## Data Availability

Not applicable.
